# Identification of Reference Genes for Quantitative RT-PCR in Ascending Aortic Aneurysms

**DOI:** 10.1371/journal.pone.0054132

**Published:** 2013-01-11

**Authors:** Dominic Henn, Doris Bandner-Risch, Hilja Perttunen, Wolfram Schmied, Carlos Porras, Francisco Ceballos, Noela Rodriguez-Losada, Hans-Joachim Schäfers

**Affiliations:** 1 Department of Thoracic and Cardiovascular Surgery, Saarland University Hospital, Homburg/Saar, Germany; 2 Department of Cardiac Surgery, Hospital Virgen de la Victoria de Malaga, Campus de Teatinos, Malaga, Spain; 3 Department of Genetics, Faculty of Biology, University of Santiago de Compostela, Santiago de Compostela, Spain; 4 Foundation IMABIS, Carlos Haya University Hospital, Avenida Carlos Haya, Malaga, Spain; The University of New South Wales, Australia

## Abstract

Hypertension and congenital aortic valve malformations are frequent causes of ascending aortic aneurysms. The molecular mechanisms of aneurysm formation under these circumstances are not well understood. Reference genes for gene activity studies in aortic tissue that are not influenced by aortic valve morphology and its hemodynamic consequences, aortic dilatation, hypertension, or antihypertensive medication are not available so far. This study determines genes in ascending aortic tissue that are independent of these parameters. Tissue specimens from dilated and undilated ascending aortas were obtained from 60 patients (age ≤70 years) with different morphologies of the aortic valve (tricuspid undilated n = 24, dilated n = 11; bicuspid undilated n = 6, dilated n = 15; unicuspid dilated n = 4). Of the studied individuals, 36 had hypertension, and 31 received ACE inhibitors or AT1 receptor antagonists. The specimens were obtained intraoperatively from the wall of the ascending aorta. We analyzed the expression levels of 32 candidate reference genes by quantitative RT-PCR (RT-qPCR). Differential expression levels were assessed by parametric statistics. The expression analysis of these 32 genes by RT-qPCR showed that EIF2B1, ELF1, and PPIA remained constant in their expression levels in the different specimen groups, thus being insensitive to aortic valve morphology, aortic dilatation, hypertension, and medication with ACE inhibitors or AT1 receptor antagonists. Unlike many other commonly used reference genes, the genes EIF2B1, ELF1, and PPIA are neither confounded by aortic comorbidities nor by antihypertensive medication and therefore are most suitable for gene expression analysis of ascending aortic tissue.

## Introduction

Ascending aortic aneurysms are associated with hypertension, connective tissue disorders [1], and congenital malformations of the aortic valve [2], [3]. The underlying pathogenetic mechanisms at the cellular level have been characterized for Marfan syndrome [1], but are still unclear for the majority of aneurysms. In hypertensive patients elevated plasma levels of matrix metalloproteinase-9 (MMP-9) have been reported, which may be the cause of increased proteolytic activity in the aortic wall and thus lead to aneurysm formation [4]. Gene expression levels in the aortic wall may additionally be influenced by antihypertensive medication of the patient. ACE inhibitors and AT1 receptor antagonists have shown to play an important role in vascular remodeling [5] which may alter the patterns of gene activities in the aortic tissue. ACE inhibitors have also been found to significantly reduce the size progression of aortic roots in patients with Marfan syndrome [6].

Increased hemodynamic stress has been proposed as the cause of aortic dilatation in patients with bicuspid aortic valves (BAV) [7], while recent studies indicate that structural deficiencies of the aortic extracellular matrix are involved in aortic dilatation [8]–[10]. Patients with unicuspid aortic valves (UAV) seem to develop aortic dilatation at an even earlier age and are also prone to the development of dissection [11]. In order to clarify the underlying molecular alterations in these aortic aneurysms, systematic investigations of the expression levels of different genes are necessary.

Gene activity analyses by RT-qPCR require the use of internal control genes with uniform activity in different samples from the given type of tissue. In many investigations, reference genes that have been employed in previous studies are used without further validation, e.g. GAPDH, beta-actin, 18S rRNA or HPRT1. These genes, however, have shown considerable variability in their expression in different tissues [12]–[15]. Thus, reference genes should be validated for each tissue type.

In order to investigate the mechanism of aortic dilatation in relation to hypertension or aortic valve morphologies, the reference genes must be independent of aortic size and aortic valve anatomy as well as the presence of arterial hypertension and antihypertensive medication. To achieve this, a panel of 32 commonly used reference genes was studied with respect to their suitability for use in RT-qPCR experiments on aortic tissue. We then analyzed the effect of hypertension, ACE inhibitors, and AT1 receptor antagonists on the expression levels of those genes which showed to be the most suitable reference genes.

## Materials and Methods

The study was conducted in accordance with the Declaration of Helsinki. All patients involved in the study have given written informed consent, and the study was approved by the locally appointed ethics committee (Ethikkommission bei der Ärztekammer des Saarlandes, No. 205/10). A total of 60 tissue specimens were obtained from the ascending aorta of patients undergoing aortic valve surgery and/or ascending aortic replacement. The valve configuration was assessed intraoperatively. Aortic size was determined by transesophageal echocardiography. The size of the ascending aorta was considered to be dilated in case of a diameter ≥40 mm. All specimens were immediately snap frozen in liquid nitrogen and further stored at −80°C.

Four patients had a UAV with dilated ascending aorta (UAVD: 4 males, mean age: 35.5 years, maximum diameter 45 to 57 mm, mean maximum diameter: 50 mm). Six had a BAV with normal ascending aorta (BAVU: 4 males, mean age: 43.5 years, maximum diameter 26 to 37 mm, mean 32 mm), 15 had a BAV with dilated ascending aorta (BAVD: 13 males, mean age: 57.4 years, maximum diameter 40 to 57 mm, mean 49 mm). In 24 a tricuspid aortic valve (TAV) with normal ascending aorta was present (TAVU: 19 males, mean age: 61.8 years, maximum diameter 26 to 39 mm, mean 33 mm), in 11 a TAV with dilated ascending aorta (TAVD: 7 males, mean age 57.4 years, maximum diameter 40 to 68 mm, mean 51 mm).

### RNA Isolation

Total RNA isolation was performed using the peqGOLD TriFast kit (Peqlab, Erlangen, Germany). For the RNA isolation frozen tissue was homogenized after addition of 1 ml TriFast (phenol and guanidinium thiocyanate) using an Ultra Turrax T8 homogenizer (Ika, Staufen, Germany). 0.2 ml chloroform per 1 ml TriFast were added in order to extract the RNA. RNA precipitation was achieved with 0.5 ml isopropanol per 1 ml TriFast and incubation on ice for no longer than 24 hours. After washing the RNA pellet with 75% ethanol, DNAse digestion and RNA cleanup were performed using the RNeasy Mini Kit (Qiagen, Hilden, Germany) according to the manufacturer’s recommendations.

### Analysis of RNA Integrity

RNA quality was determined by photometrical analysis using 1 µl of RNA sample on a Nanodrop D-1000 spectrophotometer (Nanodrop, Wilmington, USA). RNA integrity was confirmed by a denaturing RNA gel electrophoresis with agarose 1% of 4 samples (one TAVD, one TAVU, one BAVD and one BAVU). The gel was run at 4V/cm over 2 h, stained with ethidium bromide, and visualized under ultraviolet light by the Quantity One software (BioRad, Hercules, USA). RNA integrity was further assessed by an Agilent 2100 BioAnalyzer and the RNA 6000 Nano Kit (Agilent, Santa Clara, USA) according to the manufacturer’s recommendations.

### cDNA Synthesis

The High Capacity cDNA Reverse Transcription Kit (Applied Biosystems, Foster City, USA) was employed for the reverse transcription reaction according to the manufacturer’s recommendations. Only samples with an OD_260/280_ ratio from 1.8 to 2.1 were analyzed. The RT-PCR was performed with 2 µg RNA in a reaction volume of 20 µl, started at a temperature of 25°C, kept for 10 min, followed by 37°C for 120 min. Afterwards the samples were heated up to 85°C for 5 min and finally cooled to 4°C. cDNA was stored at −20°C.

### Real-time Quantitative PCR (RT-qPCR)

The expression levels of 32 commonly used candidate reference genes (Table 1) were determined using TaqMan human endogenous control plates (Applied Biosystems). These plates were analyzed using StepOnePlus Real-Time PCR Systems (Applied Biosystems), and the received data were processed using the StepOnePlus Software v2.1 (Applied Biosystems). An amount of 25 ng cDNA was used for each reaction on the 96-well optical plates. The total reaction volume of 10 µl per well contained 5 µl cDNA and DNase free water and 5 µl TaqMan Gene Expression Master Mix. Standard cycling conditions were used as recommended by the manufacturer [95°C for 10 min, (95°C for 15 seconds, 60°C for 1 min)×40 cycles].

**Table 1:** List of TaqMan Gene Assay IDs.

18S	Hs99999901_s1
GAPDH	Hs99999905_m
HPRT1	Hs99999909_m1
GUSB	Hs99999908_m1
ACTB	Hs99999903_m1
B2M	Hs99999907_m1
HMBS	Hs00609297_m1
IPO8	Hs00183533_m1
PGK1	Hs99999906_m1
RPLP0	Hs99999902_m1
TBP	Hs99999910_m1
TFRC	Hs99999911_m1
UBC	Hs00824723_m1
YWHAZ	Hs00237047_m1
PPIA	Hs99999904_m1
POLR2A	Hs00172187_m1
CASC3	Hs00201226_m1
CDKN1A	Hs00355782_m1
CDKN1B	Hs00153277_m1
GADD45A	Hs00169255_m1
PUM1	Hs00206469_m1
PSMC4	Hs00197826_m1
EIF2B1	Hs00426752_m1
PES1	Hs00362795_g1
ABL1	Hs00245445_m1
ELF1	Hs00152844_m1
MT-ATP6	Hs02596862_g1
MRPL19	Hs00608519_m1
POP4	Hs00198357_m1
RPL37A	Hs01102345_m1
RPL30	Hs00265497_m1
RPS17	Hs00734303_g1

### Statistical Evaluation

To determine the most stable reference genes among the 32 candidates, we first employed the Visual Basic Application (VBA) geNorm for Microsoft Excel [16].

In order to differentiate the effects of valve type and dilatation on gene expression, we performed a nested or hierarchic experimental design defined as follows:
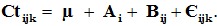



Ct_ijk_ is the Ct value in each RT-qPCR incorporating the random effect of valve type (i) and dilatation or non-dilatation (j).

**A_i_** is the random effect of each valve type on the expression of the reference genes. **B_ij_** indicates the random effect of dilatation (j) within each valve type (i), 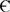
ijk is the experimental error. Additionally, the non-parametrical Kruskal-Wallis test was used to compare the Ct values of the five experimental groups (valve morphology and dilated vs. normal aorta). Kolmogorov-Smirnov test and Levene’s test were employed in order to investigate whether the gene expression showed normal distribution and fulfilled homoscedasticity. Assumptions were seen as fulfilled if p values >0.15 were obtained. An exclusion criterion was a C.V. value ≥7.0.

As a second step, the reference genes that fulfilled all of the above criteria were examined regarding the effects of hypertension, ACE inhibitors and AT1 receptor antagonists. Again, we performed a nested ANOVA with **A_i_** as the random effect of blood pressure (hypertension vs. no hypertension) on gene expression and **B_ij_** as the random effect of medication (j) within blood pressure (i). Furthermore, the Ct values of the four groups (persons with vs. without hypertension, each with vs. without ACE inhibitor/AT1 receptor antagonist) were compared using the Kruskal-Wallis test. All statistical tests were performed according to Sokal and Rohlf [17] using SPSS Statistics 19 (SPSS, Chicago, USA).

## Results

**Figure 1:**
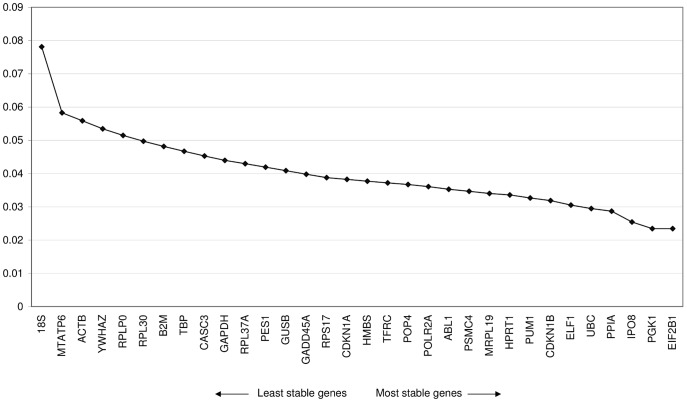
Average expression stability values of the candidate reference genes according geNorm (Method: stepwise exclusion of the least stable genes).

The results of the geNorm analysis are shown in Figure 1. The genes with the best average expression stability were EIF2B1 and PGK1 (M = 0.023), followed by IPO8, PPIA, UBC, and ELF1.

Further analyses by the Kolmogorov-Smirnov test and Levene’s test showed that not all reference genes followed a normal distribution of Ct values, or fulfilled homoscedasticity regarding valve anatomy and aortic dilatation (Table 2). Violations of homoscedasticity were shown for 19 genes by the Levene’s test, normal distribution was not present in 3 genes according to the Kolmogorov-Smirnov test. Nested ANOVA yielded effects of valve type on gene expression in 2 genes and effects of dilatation within the valve types for 10 genes. The Kruskal-Wallis test showed differences in the mean Ct value across the 5 experimental groups for 8 genes (Table 2).

**Table 2:** Descriptive statistics and tests for the investigated reference genes.

	TAVU (n = 24)	TAVD (n = 11)	BAVU (n = 6)	BAVD (n = 15)	UAVD (n = 4)	C.V.	Kolmogorov-Smirnov	Levene’s test	Nested ANOVA		Kruskal-Wallis
									A_i_	B_ij_	
Gene Symbol	M ± SD	M ± SD	M ± SD	M ± SD	M ± SD	%	p	p	p	p	p
18S	13.53±0.49	19.67±4.91	12.79±0.52	17.83±5.26	19.81±8.95	31.65	.018	.000	.320	.041	.173
GUSB	30.13±0.70	31.78±1.08	29.39±0.52	30.15±2.69	30.41±2.41	6.02	.522	.136	.361	.256	.165
CDKN1A	32.23±2.28	31.78±1.43	31.95±2.33	31.21±1.63	29.77±0.80	6.25	.725	.018	.782	.212	.151
HMBS	34.56±1.69	34.88±1.07	34.07±1.73	33.62±1.66	33.28±1.63	4.73	.785	.519	.343	.232	.208
ACTB	24.36±1.32	25.38±3.03	23.82±0.16	24.96±2.65	25.55±3.27	9.14	.593	.082	.723	.594	.968
ABL1	31.90±1.98	31.53±1.39	30.91±1.92	30.56±1.51	29.52±1.68	5.89	.360	.192	.229	.419	.083
HPRT1	31.16±0.75	32.28±1.84	30.82±1.15	32.18±2.24	31.79±2.04	5.30	.175	.153	.946	.283	.666
MRPL19	30.70±0.65	31.79±1.89	30.01±0.50	30.88±2.28	30.70±1.55	5.14	.130	.161	.539	.384	.569
B2M	24.72±1.02	27.30±1.30	24.10±1.26	26.33±2.66	26.15±2.80	8.26	.244	.127	.610	.033	.043
CDKN1B	28.60±0.54	29.66±1.29	27.68±0.72	28.54±2.20	28.50±2.46	5.61	.657	.018	.379	.404	.342
CASC3	31.64±3.24	31.26±1.83	31.30±3.02	30.62±2.09	28.62±1.61	8.64	.427	.019	.962	.069	.221
MTATP6	20.95±0.69	22.44±0.57	21.13±0.77	21.23±1.39	21.47±1.30	5.09	.969	.346	.536	.058	.166
GAPDH	26.86±1.92	27.51±1.79	26.09±0.82	27.24±2.66	27.13±2.65	7.14	.344	.192	.926	.377	.785
PES1	29.62±0.44	31.47±1.24	28.61±0.78	30.10±1.14	30.32±1.92	4.38	.579	.023	.072	.007	.047
EIF2B1	30.33±0.57	31.41±1.17	29.97±0.69	30.46±1.80	30.52±1.78	4.28	.192	.306	.544	.328	.412
ELF1	28.49±0.88	29.67±1.50	27.56±1.03	28.90±1.85	28.39±2.08	5.32	.278	.594	.431	.194	.342
GADD45A	31.35±0.67	32.42±1.97	30.43±0.50	31.28±2.72	32.23±3.04	6.37	.424	.044	.433	.558	.556
PGK1	27.90±0.77	29.37±1.22	27.66±0.68	28.32±1.52	28.44±1.62	4.47	.561	.251	.504	.104	.338
POLR2A	31.66±1.82	31.19±0.89	31.12±2.01	30.31±1.42	29.39±1.34	5.42	.424	.054	.082	.168	.080
POP4	31.26±0.76	31.89±1.40	30.79±0.75	31.25±1.53	30.67±1.48	3.90	.420	.672	.390	.590	.549
PPIA	26.05±0.68	27.35±1.37	25.61±0.63	26.62±2.29	26.62±1.81	5.90	.434	.308	.700	.250	.403
PSMC4	30.70±0.66	31.82±2.01	30.50±0.51	31.35±1.76	30.56±1.58	4.62	.147	.025	.685	.296	.690
PUM1	29.14±0.49	30.09±1.78	28.61±0.55	29.43±2.19	29.43±2.51	5.56	.262	.018	.733	.496	.673
RPL30	29.35±1.00	32.52±0.54	28.80±0.32	30.92±2.96	29.79±2.57	7.28	.288	.096	.392	.013	.026
RPL37A	26.71±0.68	29.16±0.85	25.72±0.68	27.56±2.25	27.73±2.66	6.73	.700	.028	.221	.020	.032
RPLP0	26.70±0.78	28.75±1.24	25.94±1.01	26.94±3.30	27.35±2.40	7.94	.240	.135	.410	.214	.124
RPS17	25.71±0.63	27.48±0.87	25.10±1.03	26.25±1.30	26.27±2.07	5.01	.349	.141	.239	.023	.045
TBP	33.74±1.99	33.18±2.66	34.33±3.68	32.98±1.79	31.60±0.52	6.71	.323	.007	.615	.658	.313
TFRC	31.47±0.90	33.20±1.30	30.82±1.03	31.71±1.58	31.53±1.37	4.35	.443	.411	.170	.052	.185
YWHAZ	33.23±1.22	33.94±1.43	31.90±2.00	32.60±1.85	32.34±2.61	5.30	.387	.484	.200	.660	.499
IPO8	29.84±0.47	31.26±1.42	29.51±0.51	30.27±1.75	30.12±2.28	4.74	.585	.009	.576	.185	.525
UBC	26.57±0.88	27.75±1.38	25.72±0.27	26.97±1.87	26.60±2.22	5.63	.602	.119	.469	.208	.379

Among the genes that fulfilled all statistical assumptions the following exhibited the lowest coefficients of variation (C.V.): POP4 (S. cerevisiae homolog of processing of precursor 4) (C.V.: 3.90), EIF2B1 (eukaryotic transcription initiation factor 2B, subunit 1) (C.V.: 4.28), HMBS (hydroxymethylbilane synthase) (C.V.: 4.73), HPRT1 (hypoxanthine guanine phosphoribosyltransferase 1) (C.V.: 5.30), YWHAZ (tyrosine 3-monooxygenase activation protein) (C.V.: 5.30), ELF1 (ets-related transcription factor 1) (C.V.: 5.32), and PPIA (peptidyl-propyl isomerase A) (C.V.: 5.90).

PGK1, IPO8, and UBC proved to be stable in the geNorm analysis. IPO8 and UBC, however, failed to be homogeneous according to the Levene’s test. Furthermore, nested ANOVA showed effects of dilatation within the valve types for PGK1. HMBS, HPRT1, POP4, and YWHAZ turned out to be stable in the nested statistical approach and had low C.V.s, yet other genes showed better average expression stability according to the geNorm analysis (Figure 1).

Taking both statistical approaches into account, EIF2B1, ELF1, and PPIA showed the highest stability. Therefore, they are the most suitable reference genes for RT-qPCR normalization in aortic tissue. Neither hypertension nor use of ACE inhibitors or AT1 receptor antagonists had an effect on the gene expression levels of these three genes (Table 3).

**Table 3:** Descriptive statistics and tests for the reference genes regarding effects of hypertension and medication.

	Hypertension ACE inhibitors or AT1 receptor blockers (n = 26)	Hypertension No ACE inhibitors No AT1 receptor blockers (n = 10)	No hypertension ACE inhibitors or AT1 receptor blockers (n = 5)	No hypertension No ACE inhibitors No AT1 receptor blockers (n = 19)	Levene’s test	Nested ANOVA		Kruskal-Wallis
						A_i_ 1	B_ij_ 2	
Gene Symbol	M ± SD	M ± SD	M ± SD	M ± SD	p	p	p	p
EIF2B1	30.55±1.43	31.05±1.82	30.47±0.45	30.35±1.24	.635	.548	.842	.798
ELF1	28.79±1.53	29.39±2.64	28.31±0.16	28.41±1.51	.173	.336	.832	.841
PPIA	26.52±1.81	27.02±2.02	26.36±0.53	26.22±1.28	.631	.538	.883	.806

1 A_i_ is the random effect of hypertension on the expression of the reference genes.

2 B_ij_ indicates the random effect of ACE inhibitors/AT1 receptor blockers (j) within hypertension (i).

## Discussion

Previous studies have shown that aortic dilatation involves changes of gene activities. These genes may be hereditary determinants like FBN1 in Marfan syndrome [1], or be involved in the pathogenesis of aortic dilatation in the context of congenital aortic valve malformations [2]. Aortic aneurysms in conjunction with bicuspid aortic valve anatomy are particularly important, because this entity is even more frequent than aortic dilatation in Marfan syndrome or in other hereditary connective tissue disorders [18]. In addition, hypertension has been found to be the most common underlying disease associated with the formation of aortic aneurysms including the ascending aorta [19]. Compared to connective tissue disorders and congenital aortic valve anomalies, even less is known about the molecular changes in hypertension-associated aneurysms, especially in the proximal portion of the aorta. Thus, further research on aneurysm pathogenesis is needed and crucially depends on exact quantification of gene expression based on reliable reference genes.

For normalization of gene expression analyses, an ideal reference gene for a certain type of tissue is insensitive to experimental conditions, samples and pathologies. Many previous studies [20], [21] have employed the geNorm algorithm [16] to determine stable reference genes for a given type of tissue. We additionally pursued a parametrical statistical approach where low variance, normal distribution and statistical stability of the CT values across sample groups does not only support the inference of housekeeping gene stability, but provides insight into the technical quality and reproducibility of the experiment [22]. This parametrical statistical approach is very robust as shown by several studies [23], [24].

In this regard EIF2B1, ELF1, and PPIA appear as the best candidates concerning ascending aortic tissue. All three genes have a low coefficient of variation, and their expression is not influenced by different valve morphologies, aortic diameter, hypertension, ACE inhibitors or AT1 receptor antagonists.

The EIF2B1 gene (OMIM 606686) encodes a GTP exchange factor involved in neuronal development. Constitutional mutations cause autosomal recessive leukencephalopathy with vanishing white matter (OMIM 603896). This disease does not cause structural alterations of blood vessels or connective tissue. The ELF1 gene (OMIM 189973) encodes a lymphoid-specific transcription factor that regulates inducible gene expression during T cell activation. No constitutional mutations of this gene are known. The PPIA gene (OMIM 123840) encodes an intracellular receptor protein involved in immunosuppression. No constitutional PPIA mutations are known. Somatic mutations of EIF2B1, ELF1, or PPIA have not been published so far, and with respect to the gene functions, they should not be expected to alter the mechanical properties of aortic tissue.

Surprisingly, the gene 18S (18S rRNA) which has been used for RT-qPCR normalization by previous authors [19], [20], shows a high coefficient of variation (C.V.: 31.65) and yields significant results in Levene’s test, Kolmogorov-Smirnov test, and nested ANOVA. Additionally, 18S proved to be the least stable gene in the geNorm analysis. It therefore cannot be regarded as an appropriate reference gene for aortic tissue.

The results of our study are derived from a limited sample size. Ideally, confirmation with more specimens should be aimed for. On the other hand, within our reasonable cohort size the results appeared unequivocal and the likelihood of a type II error is low.

The genes we have identified are suitable reference genes for molecular investigations of the pathogenesis of ascending aortic aneurysms. This is particularly important for investigations dealing with bicuspid and unicuspid aortic valves, which are the most frequent congenital anomalies.
